# Effects of high-dose glucocorticoids on gut microbiota in the treatment of Graves’ ophthalmopathy

**DOI:** 10.1128/spectrum.02467-24

**Published:** 2025-04-22

**Authors:** Xinhuan Su, Zhenyu Tian, Yalun Fang, Shengnan Zhou, Shizhan Ma

**Affiliations:** 1 Department of Endocrinology, Department of Geriatrics, Shandong Provincial Hospital affiliated to Shandong First Medical University 34708 , Jinan, Shandong, China; 2 State Key Laboratory for Innovation and Transformation of Luobing Theory, Key Laboratory of Cardiovascular Remodeling and Function Research of MOE, NHC, CAMS and Shandong Province, Department of Cardiology, Qilu Hospital of Shandong University, Jinan, Shandong, China; 3 Department of Clinical Laboratory, Qilu Hospital (Qingdao), Cheeloo College of Medicine, Shandong University, Qingdao, China; 4 Department of Cardiology, Shandong Provincial Hospital Affiliated to Shandong First Medical University, Jinan, Shandong, China; 5 Department of Endocrinology and Metabolism, Shandong Provincial Hospital affiliated to Shandong First Medical University, Jinan, Shandong, China; Lerner Research Institute, Cleveland, Ohio, USA

**Keywords:** high-dose glucocorticoids, Graves' ophthalmopathy, gut microbiome, short-chain fatty acids, 5-hydroxytryptamine

## Abstract

Many studies indicate the gut microbiome is associated with diseases caused by administering high-dose glucocorticoids (GCs), such as hypertension, hyperglycemia, and osteoporosis. However, the association between intestinal flora and the use of high-dose GCs remains elusive. We aimed to characterize gut microbiome in Graves’ ophthalmopathy (GO) patients after administering high-dose GCs. In this study, 20 primary GO patients were recruited. The differences in gut microbiota of GO patients before and after administering high-dose GCs were analyzed by 16S rDNA sequencing technology. Untargeted metabolomic analysis was used to examine the differences in gut metabolites between two groups. There were significant differences in α and β diversities of gut microbiota in GO patients before and after administering high-dose GCs. The random forest analysis indicated that three intestinal bacteria (*Faecalibacterium, Streptococcus,* and *Prevotella*) could distinguish the two groups with the highest accuracy, which was proven by receiver operator characteristic curve and linear discriminant analysis effect size analysis. The short-chain fatty acid-producing ability in GO patients’ gut after high-dose GC administration was significantly decreased. The 5-hydroxytryptamine levels significantly increased in the gut of GO patients after administering high-dose GCs. Our study suggests that high-dose GC administration causes the changes in gut microbiome and metabolites. Moreover, the altered flora and metabolites are related to hypertension, hyperglycemia, and osteoporosis. These findings can help understand the development of side effects caused by high-dose GCs and can be further used to develop potential probiotics to facilitate the prevention for those side effects.

**IMPORTANCE:**

For the first time, we revealed that gut microbiome and metabolome in Graves’ ophthalmopathy patients after high-dose glucocorticoid (GC) administration significantly changed, and the altered flora and metabolites are related to hypertension, hyperglycemia, and osteoporosis. These findings can help understand the development of side effects caused by high-dose GCs and can be further used to develop potential probiotics to facilitate the prevention for those side effects.

## INTRODUCTION

Glucocorticoids (GCs) are commonly utilized in various clinical states due to their potent anti-inflammatory and immunosuppressive properties. About 1%–1.2% of the general population undergoes systemic therapy with GCs at some point in their lives, with the course of treatment usually lasting for months or years (1). However, long-term or high-dose application of GCs can lead to metabolic disorders, including abdominal obesity, hyperglycemia, hypertension, and more (2, 3), which severely limits their clinical application. The mechanism of GCs-induced metabolic disorder is not yet fully understood.

Graves’ ophthalmopathy (GO), also known as thyroid-associated ophthalmopathy, is an organ-specific autoimmune disease. It ranks first in the incidence of adult orbital diseases and is the most common extrathyroid manifestation of Graves’ disease (GD), occurring in 25% to 40% of GD patients. GO is characterized by inflammation, infiltration, and edema of the eye tissues, which, in severe cases, can lead to vision impairment or even blindness, significantly affecting the patient’s quality of life (4). The treatment of GO includes medication, orbital radiotherapy, and surgery. Among them, drug therapy mainly includes high-dose GCs, biological agents, and traditional immunosuppressants. High-dose GC therapy is an important treatment for GO, especially in controlling acute inflammatory responses (5).

As technology continues to advance, the intricate relationship between the human microbiome, especially the gut microbiome, and a range of associated diseases has been increasingly uncovered and been the subject of extensive research (6). The gut microbiota is essential for maintaining the host’s physiological functions by regulating body metabolism and immunity (7, 8). Imbalances in the gut microbiota and its metabolites are closely related to metabolic syndrome and diabetes (9). To date, studies have found that obvious gut microbiota dysbiosis was observed in patients with endogenous hypercortisolism (10, 11).

The therapeutic use of GCs is limited by a wide range of side effects, such as osteoporosis, hyperglycemia, insulin resistance, disturbed fat deposition, hypertension, and muscle atrophy (12, 13). Indeed, GCs were empirically used in the clinic before elucidation of their mechanisms of action. The precise cellular mechanisms underpinning the differential action of GCs to drive the desirable and undesirable effects are still not completely understood (13). At the same time, high-dose GCs is widely used in GO patients, while its potential impact on the gut microbiota has not been reported. To date, only one human study has analyzed the gut microbiota in 28 patients with acute transverse myelitis who received prednisone (50 mg–60 mg/day) for 7 days, which found a significant reduction in gut microbial diversity, with an increase in *Firmicutes* and a decrease in *Bacteroidetes* in GC-induced obese individuals (14). The newly published study conducted on mice has found that gut microbiota dysbiosis and decreased levels of serum acetic acid and propionic acid may participate in GC-induced glycolipid metabolism disorder (15). Therefore, exploring the changes in the gut microbiota of GO patients after high-dose GC therapy is of great clinical significance. This not only helps to better understand the effects of high-dose GCs on the gut microbiome but also provides novel insights into the prevention and treatment of GC-induced side effects.

As an exploratory study, this research aimed to analyze the effects of high-dose GC administration on intestinal flora in GO patients by sequencing the 16S ribosomal RNA (rRNA) gene for the first time. We also examined the gut metabolome to observe the functional changes of gut microbiota in GO patients after high-dose GC treatment. Our study indicates that high-dose GC administration leads to alterations in the gut microbiome and metabolome. Moreover, the changes in the gut microbiome and metabolome are associated with side effects of high-dose GC administration, including hypertension, hyperglycemia, and osteoporosis. These findings provide insights into the mechanisms underlying side effects induced by high-dose GCs and may contribute to the development of potential probiotic interventions to prevent such adverse effects.

## MATERIALS AND METHODS

### Study cohort and recruitment of participants

We recruited 20 primary GO patients in our study at the Shandong Provincial Hospital. All primary GO patients were diagnosed based on laboratory tests and imaging examination, and none had received medical treatment for almost 6 months. Then, methylprednisolone intravenous pulse therapy was applied to them. The cumulative dose is 4.5 g over 12 weeks. The regimen is as follows: 0.5 g of methylprednisolone administered via intravenous infusion once a week for 6 weeks, followed by 0.25 g of methylprednisolone administered via intravenous infusion once a week for another 6 weeks (16, 17). The following exclusion criteria were applied to all patients: pregnancy; smoking; alcohol addiction; diabetes mellitus; recent (within the past 3 months) use of antibiotics, probiotics, prebiotics, symbiotics, hormonal medication, laxatives, proton pump inhibitors, or traditional Chinese medicine; a known history of autoimmune-related diseases, such as multiple sclerosis (MS), rheumatoid arthritis, irritable bowel syndrome, or inflammatory bowel disease; as well as any history of malignancy or gastrointestinal surgery.

### Sample collection

Fresh stool samples from GO patients were collected before and after the administration of high-dose GCs. Fecal samples were collected by the patients using disposable sterile forceps in the morning. Basic information, such as the collection time and patient name, was recorded on the sample collection container. After collection, each fecal sample was immediately divided into aliquots and stored at −80°C until DNA extraction.

### 16S rRNA gene sequencing

Total genome DNA from the fecal samples was extracted using the improved cetyltrimethylammonium bromide method (18). We used Nanodrop 2000 (Thermo Scientific) spectrophotometer to determine the concentration of the extracted DNA. The V3-V4 regions of the 16S rRNA gene were amplified and sequenced on an Illumina NovaSeq 6000 system. The PCR was conducted using the bacterial universal primers (Univ 337F/Univ 806R) (Table S1) (19). Then, the amplicons were sequenced on an Illumina NovaSeq 6000 system and 410 bp paired-end reads were generated.

### Bioinformatic analysis of sequencing data

The raw sequencing data were merged and quality filtered using Laser FLASH method, described by Magoč and Salzberg (20). Paired-end reads were assigned to each sample according to the unique barcodes. The 16S rRNA operational taxonomic units were selected from the combined reads using the QIIME2 (Quantitative Insights into Microbial Ecology) software package (http://qiime2.org/) and were annotated for taxonomic information by the Silva 138 ribosomal database. Core_diversity_analyses.py scripts were used to analyze α diversity (within samples) and β diversity (among samples). The linear discriminant analysis (LDA) effect size (LEfSe) analysis was performed to identify differentially abundant bacterial taxa as biomarkers (LDA score >2 and *P* < 0.05) (21). Kyoto Encyclopedia of Genes and Genomes (KEGG) pathways and clusters of orthologous groups functions were analyzed using PICRUSt software (22, 23). Spearman correlation was calculated using R package psych. To assess whether taxonomic differences in the microbiota could classify samples into different cohorts and predict outcomes, a machine learning algorithm called Random Forest (RF) was used to analyze genus-level abundances of gut bacteria, utilizing the R package randomForest (24). We conducted a receiver operating characteristic (ROC) analysis to evaluate the performance of metagenomic biomarkers after high-dose GC treatment, using the “pROC” package in R software (24). Figures were mainly painted by R package “ggplot2” (v.3.1.0).

### Untargeted metabolomic analysis of stool samples

The LC/MS system for metabolomics analysis is composed of Waters Acquity I-Class PLUS ultra-high performance liquid tandem Waters Xevo G2-XS QTof high resolution mass spectrometer. Waters Xevo G2-XS QTof high resolution mass spectrometer can collect primary and secondary mass spectrometry data in MSe mode under the control of the acquisition software (MassLynx v.4.2, Waters). The parameters of the Electrospray Ionization (ESI) ion source are as follows: capillary voltage: 2,000V (positive ion mode) or −1,500V (negative ion mode); cone voltage: 30V; ion source temperature: 150°C; desolvent gas temperature 500°C; backflush gas flow rate: 50 L/ h; desolventizing gas flow rate: 800 L/h. The raw data collected using MassLynx v.4.2 is processed by Progenesis QI software for peak extraction, peak alignment, and other data processing operations, based on the Progenesis QI software online METLIN database and Biomark’s self-built library for identification, and at the same time, theoretical fragment identification and mass deviation. All are within 100 ppm. After normalizing the original peak area information with the total peak area, the follow-up analysis was performed. Principal component analysis and Spearman correlation analysis were used to judge the repeatability of the samples within group and the quality control samples. The identified compounds are searched for classification and pathway information in KEGG, The Human Metabolome Database (HMDB), and lipid maps databases.

### Quantitative real-time PCR

The genomic DNA (gDNA) from the fecal samples was extracted using a previously described method. Then, SYBR Green qPCR technology was employed to analyze the changes in the phyla of *Firmicutes*, *Bacteroidetes*, as well as the genes of ButA (butyryl-CoA transferase), LcdA (lactoyl-CoA dehydratase), PduP (propionaldehyde dehydrogenase), and MmdA (methylmalonyl-CoA decarboxylase). Then, quantitative PCR was carried out using TB Green Premix Ex Taq (Takara) and specific primers (Table S1) on a CFX96 Real-Time PCR System (Bio-Rad, USA). Relative gene expression was calculated by the 2^-∆∆Ct^ method using 16S rRNA gene (Univ 337F/Univ 518R) as an internal control (Table S1).

### Statistical analysis

The paired samples Wilcoxon test, with appropriate correction for multiple comparisons, was employed to identify significant differences in continuous outcomes. *P* < 0.05 was considered as statistically significant. For certain analyses, a false discovery rate corrected *P*-value (q-value) <0.05 was considered statistically significant. All analyses were performed using R software (version 4.4.0), and GraphPad Prism 8 software.

## RESULTS

### Gut microbiota of GO patients underwent significant changes after the administration of high-dose GCs

**Fig 1:**
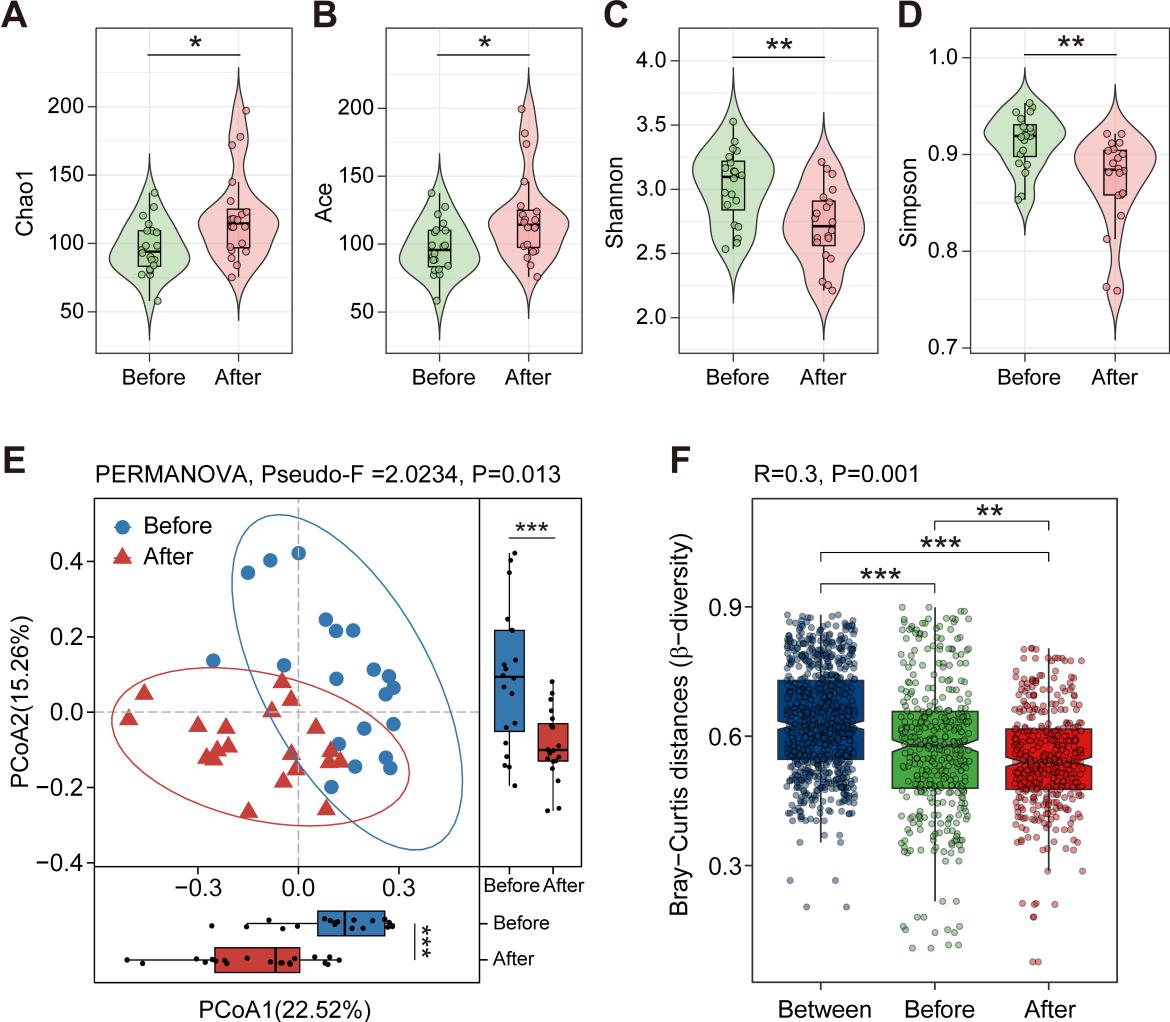
Composition of gut microbiota is altered significantly in GO patients after administering high-dose GCs. (**A–D**) The α diversity indices (Chao1, ACE, Shannon, and Simpson indices) of the intestinal flora in GO patients before and after administering high-dose GCs. The paired samples Wilcoxon test was used to detect significant changes. (**E**) A principal component (PCoA) score plot based on Bray-Curtis distance matrix for all participants. Each point represents the composition of the intestinal microbiota of one participant. The ellipses do not represent statistical significance but rather serve as a visual guide to group diﬀerences. The PERMANOVA statistical test was used to determine whether the clustering effect in the PCoA was significant, and the paired samples Wilcoxon test was employed to compare the differences between groups for the first and second principal components of the PCoA. (**F**) Similarity analysis (ANOSIM) revealed differences in microbial community structure between groups before and after high-dose GC administration. *, *P* < 0.05; **, *P* < 0.01; ***, *P* < 0.001. Before, GO patients before administering high-dose GCs; After, GO patients after administering high-dose GCs. *n*_Before_ = 20; *n*_After_ = 20.

In this study, 20 initial GO patients were recruited and the stool samples were collected before and after the administration of high-dose GCs. The demographic details of all participants are provided in Table S2. To clarify the effect of high-dose GCs on the gut microbiota of GO patients, fecal samples were analyzed through 16S rRNA gene sequencing. The sequencing depth was saturated and was not significantly different between the two groups of GO patients before and after administering high-dose GCs (Fig. S1A through C). The Chao1 and Abundance-based Coverage Estimator (ACE) indices, which represent the richness of the gut microbiota, showed a significant increase in GO patients after the administration of high-dose GCs (Fig. 1A and B). However, the Shannon and Simpson indices, which indicate gut microbiota diversity, were significantly reduced in GO patients after high-dose GCs administration (Fig. 1C and D). All the findings suggest that GO patients experienced an increase in gut microbiota richness but a decrease in community diversity following high-dose GC administration. The Analysis of Similarities (ANOSIM) and Bray-Curtis distance-based community analysis revealed a clear separation in the gut microbiota characteristics of GO patients before and after high-dose GC administration, indicating a significant difference in microbiota composition between the two groups (Fig. 1E and F).

**Fig 2:**
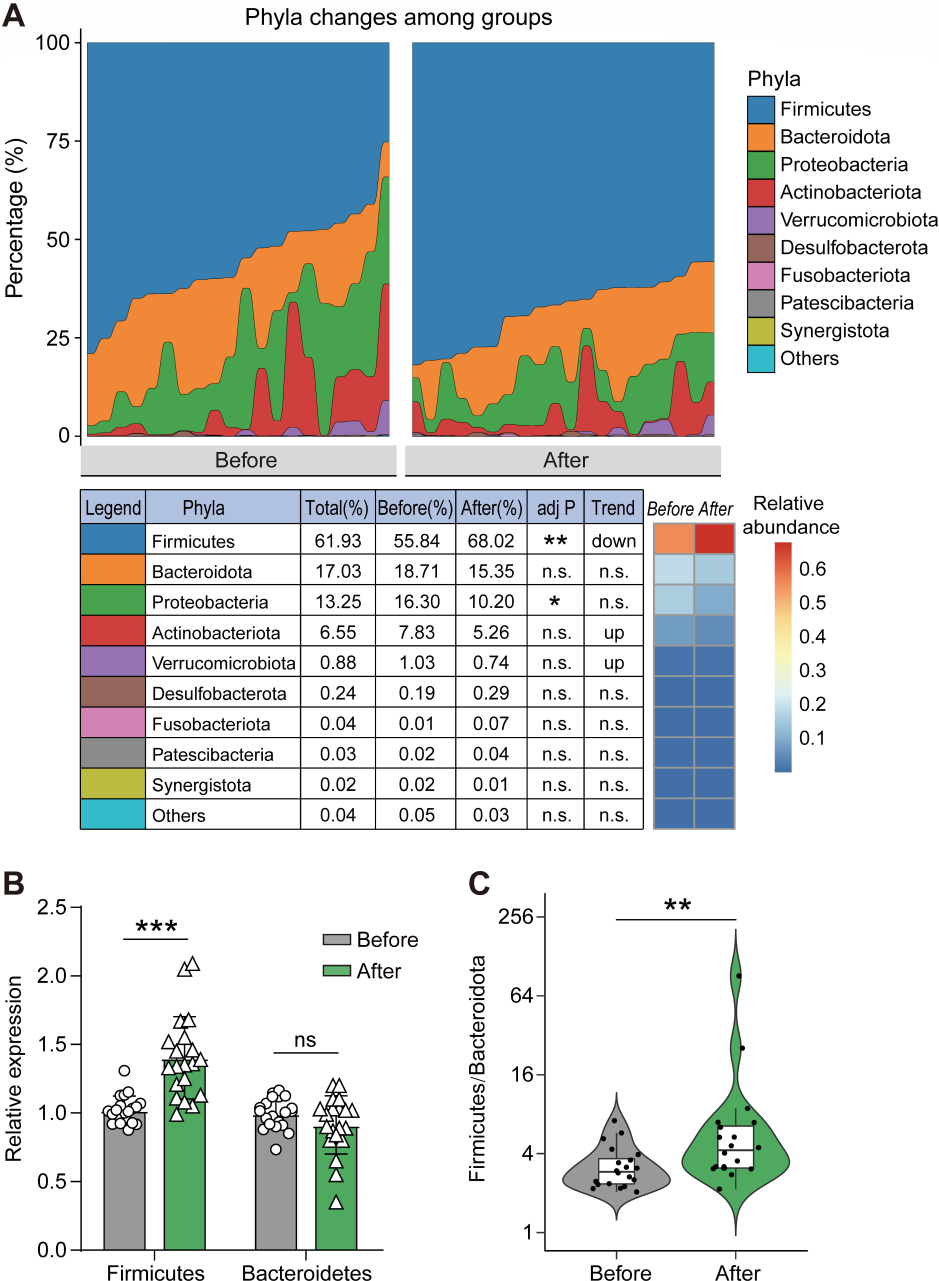
Gut microbiota of GO patients after administering high-dose GCs significantly changed at phylum level. (**A**) The relative abundances of main intestinal bacteria in GO patients before and after administering high-dose GCs and comparisons of the intestinal flora of the two groups at the phylum level. (**B**) The fold changes in the abundances of *Firmicutes* and *Bacteroidetes* in the intestinal flora of GO patients before and after administering high-dose GCs. They were determined by qPCR. (**C**) The ratio of *Firmicutes*/*Bacteroidetes* in GO patients before and after administering high-dose GCs was calculated using the 16S rRNA gene sequences data. The paired samples Wilcoxon test was used to detect significant changes. ns, not significant; *, *P* < 0.05; **, *P* < 0.01; ***, *P* < 0.001. Before, GO patients before administering high-dose GCs; After, GO patients after administering high-dose GCs. *n*_Before_ = 20; *n*_After_ = 20.

At the phylum level, there was no difference in composition between the two groups, with over 90% of the bacteria predominantly consisting of *Firmicutes*, *Bacteroidetes*, and *Proteobacteria* (Fig. 2A). The abundance of *Firmicutes* increased in GO patients after administering high-dose GCs compared to before treatment, while the abundance of *Bacteroidetes* (not statistically significant), *Actinobacteriota* (not statistically significant), and *Proteobacteria* decreased (Fig. 2A), which was further validated through quantitative PCR (qPCR) (Fig. 2B). As a result, the *Firmicutes*/*Bacteroidetes* (F/B) ratio significantly increased in GO patients following high-dose GC administration (Fig. 2C).

**Fig 3:**
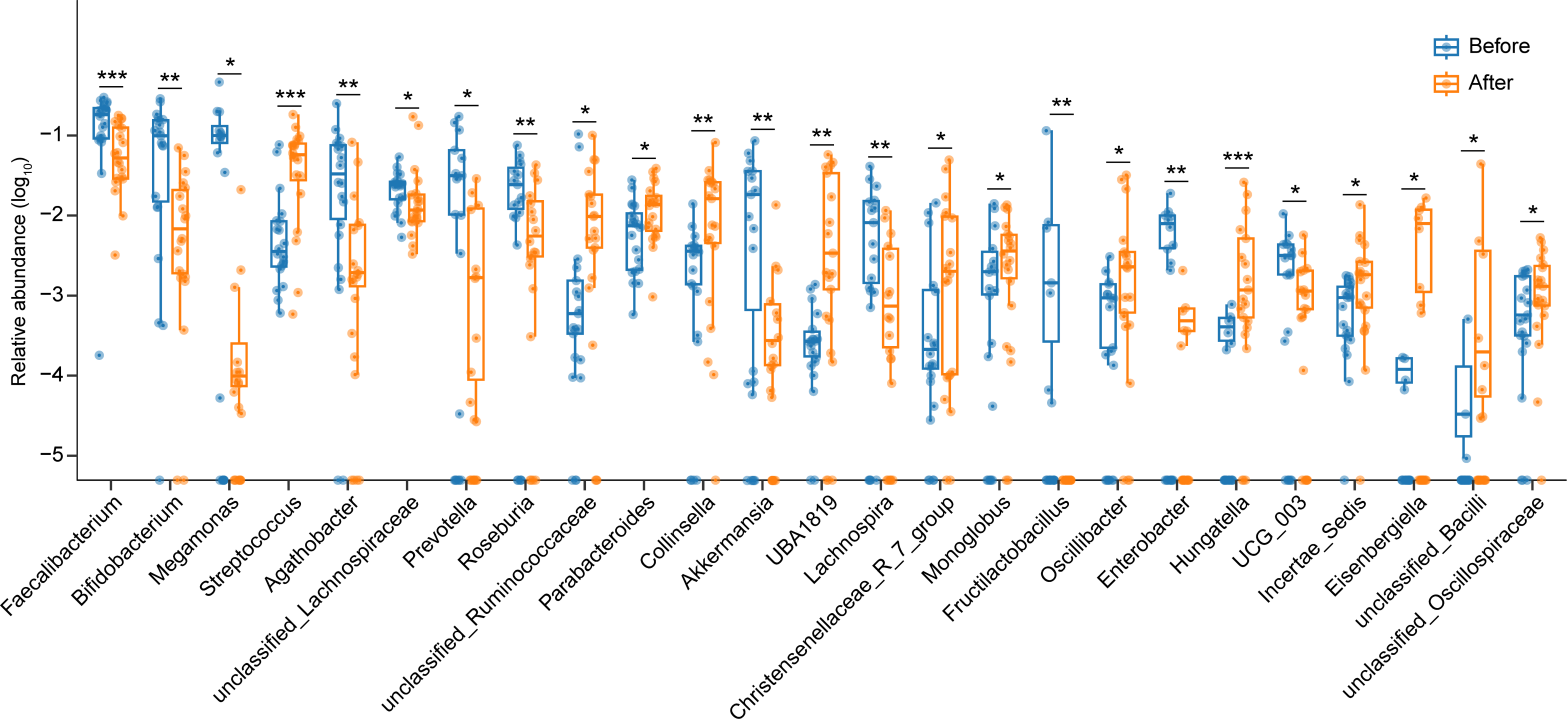
Gut microbiota of GO patients after administering high-dose GCs significantly changed at genus level. Significantly altered gut bacteria in GO patients after administering high-dose GCs at the genus level with abundance greater than 0.001%. The paired samples Wilcoxon test was used to detect significant changes. *, *P* < 0.05; **, *P* < 0.01; ***, *P* < 0.001. Before, GO patients before administering high-dose GCs; After, GO patients after administering high-dose GCs. *n*_Before_ = 20; *n*_After_ = 20.

**Fig 4:**
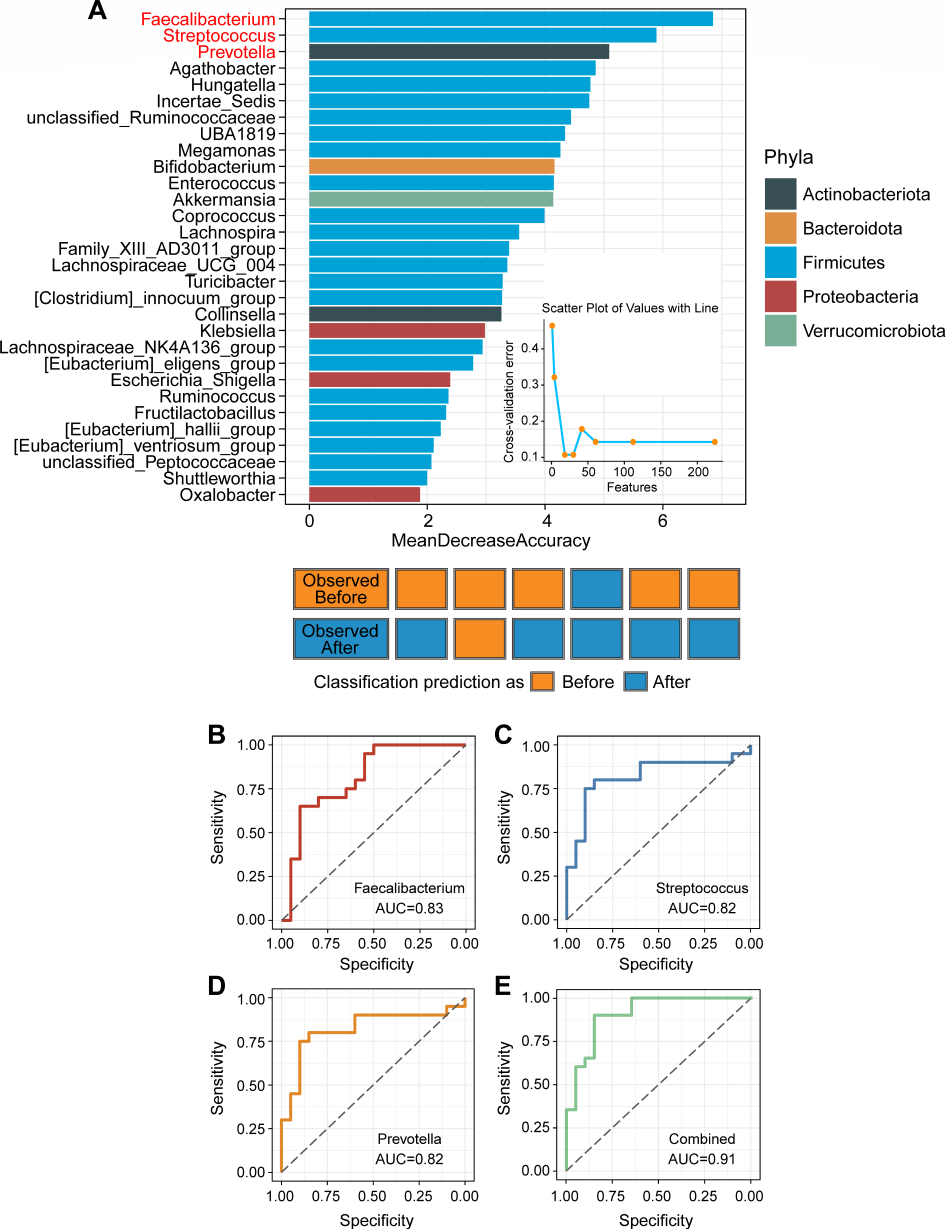
RF and ROC curves analysis. (**A**) The top 30 important gut microbial signatures identified by the Random Forest algorithm in the training set of GO patients before and after administering high-dose GCs. Three bacterial genera that could accurately distinguish GO patients before and after administering high-dose GCs with the highest accuracy are indicated in red font. Classifier accuracy per person in the test set revealed by the Random Forest algorithm. (**B–D**) Accuracy of the three gut bacteria-based RF predictive model is measured by AUC in GO patients before and after administering high-dose GCs. B, *Faecalibacterium*; C, *Streptococcus*; D, *Prevotella*. (**E**) ROC curve of the RF model using the relative abundances of the three genera together.

**Fig 5:**
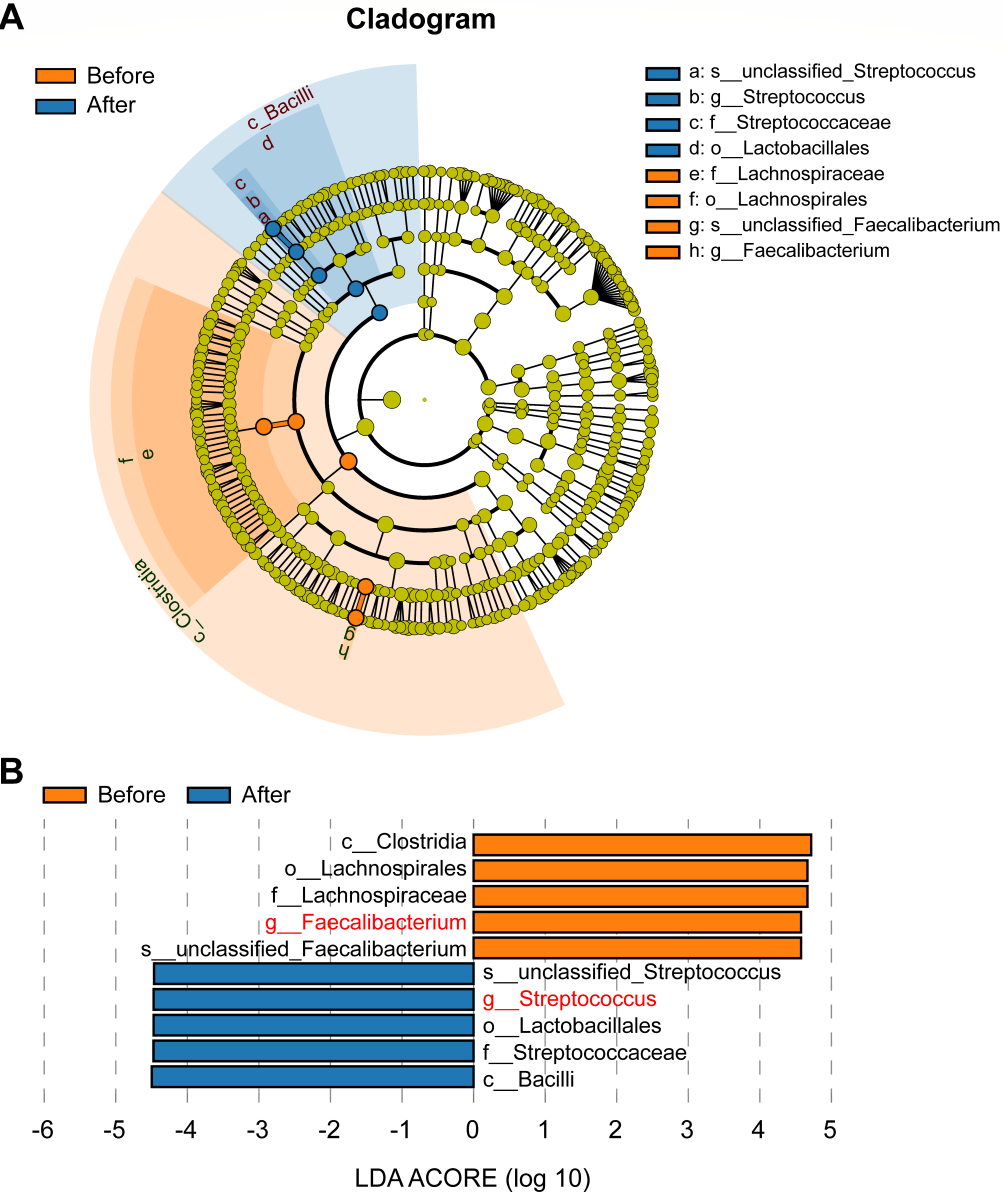
Differentially abundant bacterial taxa between GO patients before and after administering high-dose GCs by LEfSe analysis. (**A**) Cladogram generated by LEfSe analysis. The significantly differential bacterial clades or taxa are highlighted. Red: increased abundance in Before group. Blue: increased abundance in After group. (**B**) LDA scores of the intestinal bacteria in GO patients before and after administering high-dose GCs at different taxonomic levels generated by LEfSe analysis. LDA score >4.5 (<−4.5) indicates that bacterial taxa are significantly enriched in Before (After) group (*P* < 0.05). The important bacteria are shown in red font. Before, GO patients before administering high-dose GCs; After, GO patients after administering high-dose GCs.

At the genus level of intestinal bacteria (abundance >0.001%), the abundances of 13 bacterial genera significantly increased, while 12 genera markedly decreased in GO patients following high-dose GC administration (Fig. 3). Among the bacteria with significant content and changes, the abundance of *Streptococcus*, *Parabacteroides*, and *Collinsella* in GO patients after high-dose GC administration significantly increased, while *Faecalibacterium*, *Bifidobacterium*, *Prevotella*, and *Akkermansia* significantly decreased (Fig. 3). Interestingly, under the application of high-dose GCs, *Fructilactobacillus* was completely inhibited and disappeared. To identify the bacterial genera most closely associated with GO patients after high-dose GC administration, we performed a series of analyses. The RF analysis revealed that three intestinal bacteria (*Faecalibacterium*, *Streptococcus*, and *Prevotella*) could distinguish GO patients after administering high-dose GCs from those before treatment with the highest accuracy (Fig. 4A). These findings indicated that the three bacteria were closely associated with the use of high-dose GCs. To evaluate the group after administering high-dose GCs based on these biomarkers, an ROC curve analysis was performed on the two-group data. The ROC results showed that the area under the receiver operating characteristic (AUC) for each of the three bacteria exceeded 80% (Fig. 4B through D), indicating that each of the three bacteria could be used to classify the group after administering high-dose GCs with high accuracy. Among the three bacteria，*Faecalibacterium* and *Prevotella* significantly reduced in the GO patients after administering high-dose GCs, while *Streptococcus* significantly increased. We then performed the ROC analysis again based on the merger of these three bacteria with 91% AUC (Fig. 4E). This further validates the results of our RF analysis. Additionally, LEfSe analysis revealed that *Streptococcus* was enriched in GO patients before high-dose GC administration, while *Faecalibacterium* was enriched in GO patients after high-dose GC administration among these biomarkers (Fig. 5A and B).

### Imputed microbiome function and the declining short-chain fatty acid (SCFA)-producing ability after administering high-dose GCs

**Fig 6:**
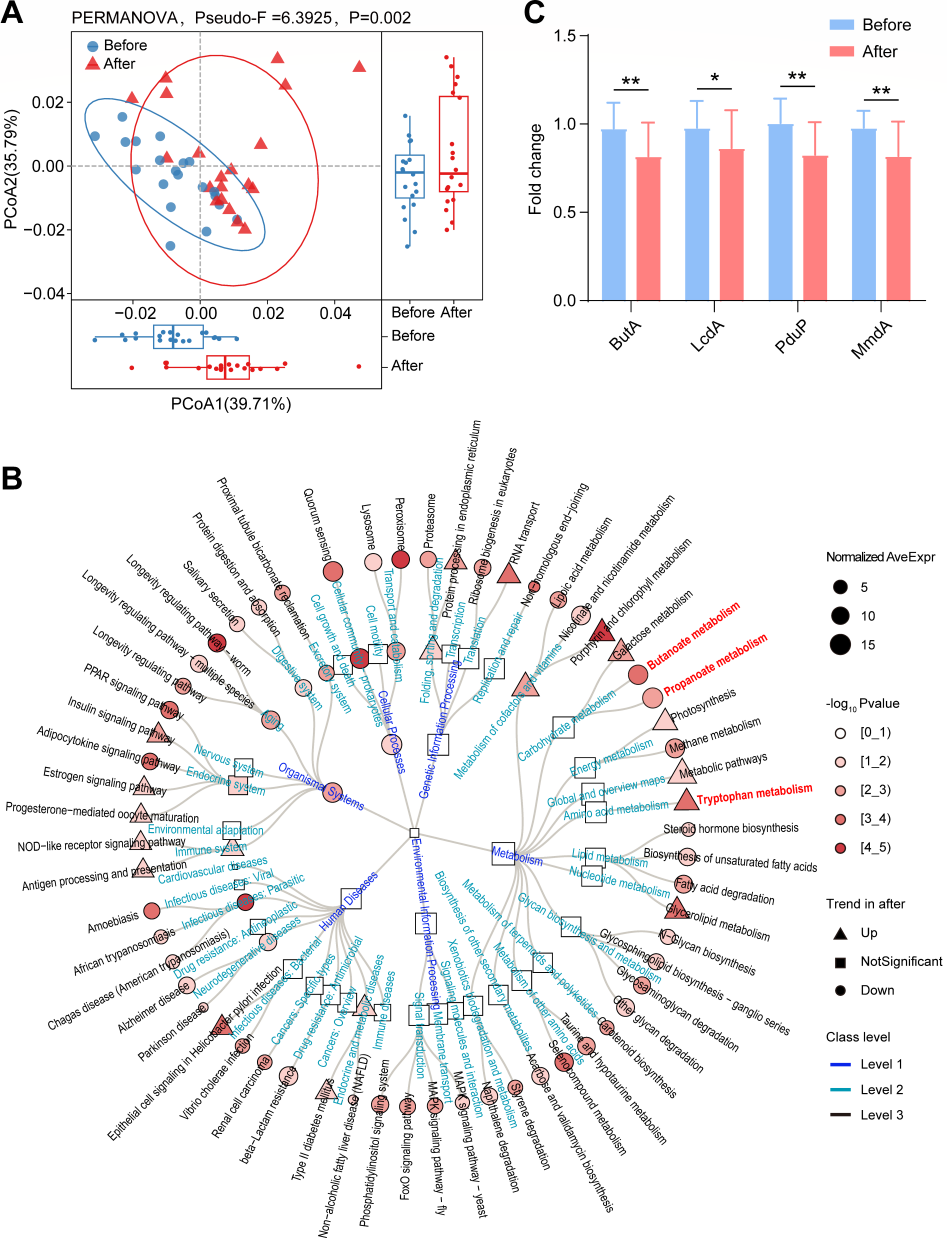
Imputed metagenomic differences between GO patients before and after administering high-dose GCs. (**A**) The relative abundance of metabolic pathways encoded in each imputed sample metagenome was analyzed for a principal component (PCoA) using R. (**B**) Phylogenetic tree analysis of imputed metagenomic through 16s rRNA sequencing data in GO patients’ gut before and after administering high-dose GCs. Class levels 1, 2, and 3 represent KEGG_A_class, KEGG_B_class, and KEGG pathway, respectively. Triangles, circles, and squares represent significant increase, significant decrease, and no difference of metagenomics functions, respectively, in GO patients after high-dose GCs treatment. The darker the color, the greater the statistical difference. In class level 3, the important metagenomics functions are indicated in red font. (**C**) The abundance changes of the key enzymes for producing butyrate and propionate in the intestinal flora of GO patients after administering high-dose GCs by qPCR. The paired samples Wilcoxon test was used to detect significant changes. *, *P* < 0.05; **, *P* < 0.01. ButA, butyryl-CoA transferase; LcdA, lactoyl-CoA dehydratase; PduP, propionaldehyde dehydrogenase; MmdA, methylmalonyl-CoA decarboxylase. Before, GO patients before administering high-dose GCs; After, GO patients after administering high-dose GCs. *n*_Before_ = 20; *n*_After_ = 20.

Considering the structural changes in the microbiome of GO patients after high-dose GC administration, we next investigated whether high-dose GCs would also induce functional changes within each microbiome. In the absence of shotgun metagenomic sequencing data, we applied PICRUSt to our 16S rRNA gene survey to predict the functional content of the metagenome. PICRUSt is a computational method that employs evolutionary modeling to predict the present gene families based on 16S rRNA data and a reference genome database. The inferred relative abundances of KEGG pathways in each sample were used to predict metabolic function changes in the microbiomes of GO patients before and after administering high-dose GCs. The Bray-Curtis distance-based community analysis showed an obvious separation, indicating that the metabolic function composition diﬀered significantly between the two groups (Fig. 6A). For KEGG_A_class, cellular processes and organismal systems had lower predicted relative abundance in GO patients after administering high-dose GCs (Fig. 6B). For KEGG_B_class, the predicted relative abundance of pathways related to endocrine and metabolic diseases, immune system, folding, sorting and degradation, and metabolism of cofactors and vitamins significantly increased in GO patients after administering high-dose GCs. In contrast, pathways associated with neurodegenerative diseases, infectious diseases— parasitic, aging, digestive system, excretory system, circulatory system, cellular community–prokaryotes, and transport and catabolism—significantly decreased (Fig. 6B). For KEGG pathway, a total of 60 metabolic pathways showed significant differences, with 15 enriched and 45 diminished in GO patients after administering high-dose GCs (Fig. 6B). Among them, the predicted relative abundance of butanoate and propanoate metabolism was lower in GO patients after administering high-dose GCs (Fig. 6B).

Several key enzymes, such as ButA, LcdA, PduP, and MmdA, are involved in the production of butyrate and propionate in the gut microbiota. We assessed the expression levels of these genes using qPCR. The abundances of these key enzyme genes significantly decreased in the gut microbiota of GO patients after administering high-dose GCs, confirming a substantial reduction in the ability to produce SCFAs in these patients following the treatment (Fig. 6C). This result also confirmed the alterations in butanoate and propanoate metabolism in GO patients following high-dose GC administration.

### Gut metabolites were significantly changed in GO patients after administering high-dose GCs

**Fig 7:**
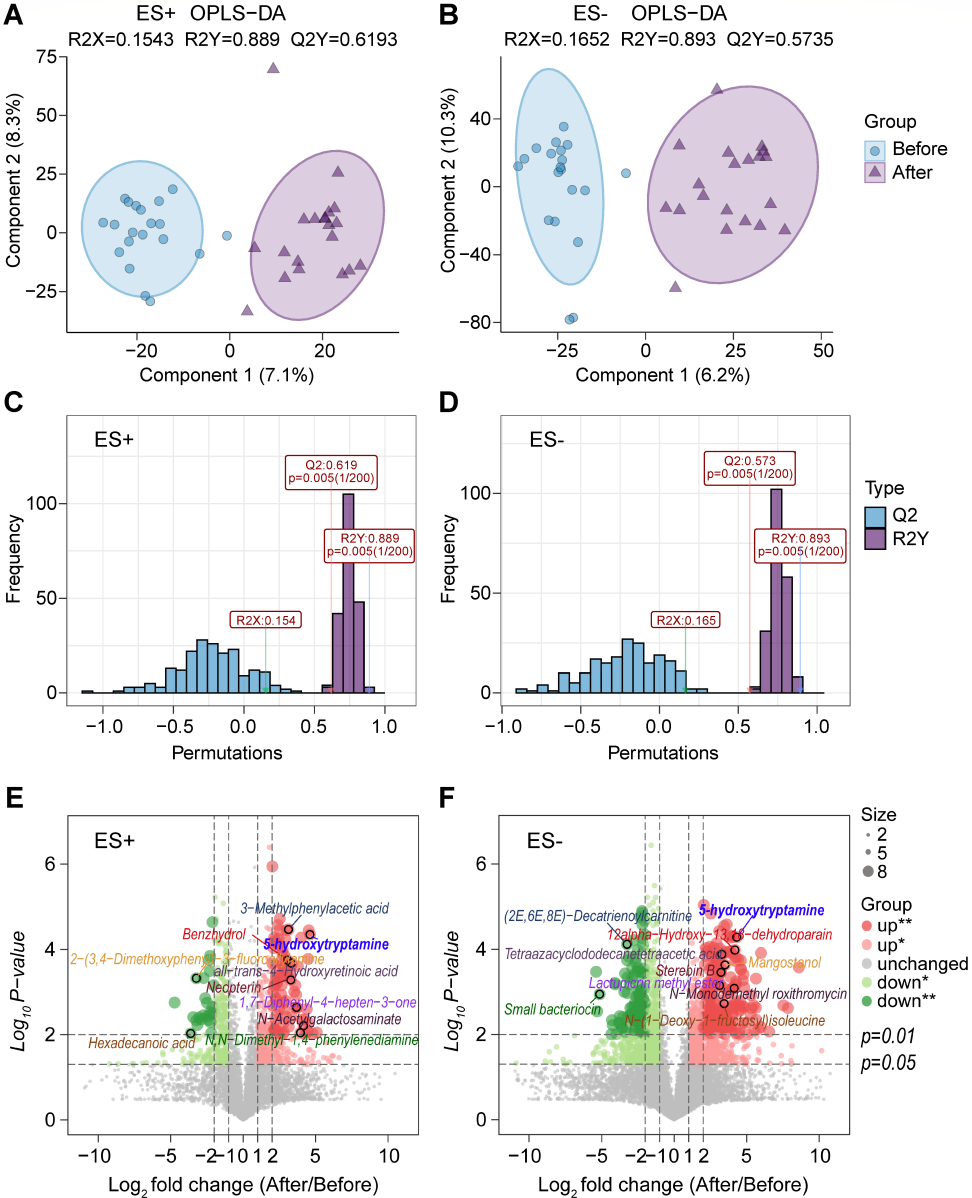
Noticeable changes in the metabolic profiles of gut microbiota in GO patients after high-dose GC administration. (**A, B**) The plot of OPLS-DA score of all peak features from the untargeted metabolomics analysis of stool samples of GO patients before (Before) and after high-dose GC administration (After) in positive ion mode (ES+) (**A**) and negative ion mode (ES−) (**B**). (**C, D**) Validation of the OPLS-DA model via permutation test (times = 200) in positive ion mode (ES+) (**C**) and negative ion mode (ES−) (**D**). (**E, F**) Changes in gut metabolites in GO patients before and after high-dose GC treatment are shown in positive ion mode (ES+) (**E**) and negative ion mode (ES−) (**F**). Red and green, respectively, indicate the increased and decreased abundance of gut metabolite in GO patients after high-dose GC treatment. The size of the circles represents the relative abundance of the metabolite. The paired samples Wilcoxon test was used to detect significant changes. *, *P* < 0.05; **, *P* < 0.01. Before, GO patients before administering high-dose GCs; After, GO patients after administering high-dose GCs. *n*_Before_ = 20; *n*_After_ = 20.

Gut metabolites are important bridges for intestinal ﬂora to regulate the body health. Therefore, metabolomic analysis was conducted to investigate the intestinal metabolic profiles of GO patients following high-dose GCs treatment. A total of 20,423 and 11,271 peak features were identified, respectively, in positive ion mode (ES+) and negative ion mode (ES−). Orthogonal partial least squares discriminant analysis (OPLS-DA) was used to cluster these peak features, revealing a clear separation between the samples taken before and after high-dose GC treatment (Fig. 7A through D). These results suggest significant alterations in the intestinal metabolic profiles of GO patients following high-dose GC treatment. The significantly altered peak features were analyzed using MS/MS, and numerous metabolites were identified by combining accurate molecular weight data with structural information from the compound structure database. The metabolites identified included amino acid derivatives, lipids, fatty acids, and bile acids, with many exhibiting significant alterations in GO patients following high-dose GC therapy (Fig. 7E and F). Among these differential metabolites, we found that the abundance of 5-hydroxytryptamine (5-HT) increased significantly in GO patients after high-dose GC administration in positive ion mode (ES+) (Fig. 7E) and negative ion mode (ES−) (Fig. 7F). This is consistent with our functional prediction that tryptophan metabolism increased after high-dose GC treatment (Fig. 6B), as tryptophan metabolism is the key pathway for 5-hydroxytryptamine synthesis. In addition, we conducted an analysis of the correlation between key gut microbiota and gut metabolites, which revealed a close association between these differential microbial taxa and metabolites in the gut (Fig. S2).

## DISCUSSION

Long-term/high-dose GC use results in metabolic disorder, but the role of gut microbiota in GC-induced metabolic dysfunction remains unknown. To date, only one human study has analyzed the gut microbiota in 28 patients with acute transverse myelitis who received prednisone (50 mg/day–60 mg/day) for 7 days (14). In our study, we firstly revealed gut microbiota and metabolite alterations in GO patients with high-dose GC treatment which lasted for 12 weeks using a total of 4.5 g of methylprednisolone. We observed the changes in gut microbiota and metabolites of GO patients during longer-term and higher-dose GC treatment.

In the composition of the gut microbiome, there was a significant difference in the α diversity of GO patients after high-dose GC administration. Firstly, the Chao1 and ACE indices were increased significantly in GO patients after high-dose GC administration, which indicated that the high-dose GCs increased community richness of gut microbiota and the gut microbiota was in a disrupted and impaired state (25), which is consistent with many disease states. Secondly, the lower Shannon and Simpson indices of the intestinal flora in GO patients after administering high-dose GCs indicated the decreased community diversity, which is consistent with a previous study (14). Furthermore, the β diversity of ANOSIM and Bray-Curtis distance-based community analysis showed an obvious separation between the two groups, revealing that administering high-dose GCs significantly impacted microbiota composition of GO patients.

In the gut of healthy individuals, the F/B ratio is relatively stable, and an increase in the F/B ratio often means an abnormal condition or a disease state (26). The F/B ratio of the GO patients after administering high-dose GCs increased, which is consistent with a previous study (14). Many diseases, like hypertension, hyperglycemia, and osteoporosis, are associated with a higher intestinal F/B ratio (27–29). The greater F/B ratio may be one reason why high-dose GC therapy is likely to result in side effects such as hypertension, hyperglycemia, and osteoporosis. However, further researches are needed to elucidate the mechanism.

For patients undergoing high-dose GC therapy in clinical practice, routine monitoring of blood pressure (BP) and blood sugar is performed, along with timely calcium supplementation and gastric mucosa protection. Although these procedures are simple and clear, we generally wait until the patient develops issues with BP or blood sugar before proceeding with further treatment interventions. Moreover, routine calcium supplementation and the use of acid-suppressing medications cannot completely prevent the occurrence of osteoporosis and gastric mucosal damage, and the quality of life of patients is greatly affected. So, the intestinal bacteria could be the new target for preventing the side effects caused by high-dose GCs. Previous research showed that the *Streptococcus* spp. was positively correlated with BP (30). Our result also showed the increased abundance of the genus *Streptococcus,* which belongs to the family *Streptococcaceae,* in GO patients after high-dose GC administration, which indicated that *Streptococcus* may be closely related to hypertension caused by high-dose GC administration. Among the commonly reported findings, the genera of *Bifidobacterium*, *Bacteroides*, *Faecalibacterium*, *Akkermansia,* and *Roseburia* were negatively associated with hyperglycemia, while the genera of *Ruminococcus*, *Fusobacterium*, and *Blauti*a were positively associated with hyperglycemia (31). Although we did not observe a significant increase in *Ruminococcus*, *Fusobacterium*, and *Blautia*, we found a marked decrease in the abundance of *Bifidobacterium*, *Faecalibacterium*, *Akkermansia*, and *Roseburia* in GO patients’ gut after high-dose GCs. So, correcting the abundance of *Bifidobacterium*, *Faecalibacterium*, *Akkermansia*, and *Roseburia* may reduce the risk of developing GC-induced hyperglycemia. Data indicated that *Veillonella*, *Parabacteroides*, and *Harryflintia* were mainly enriched in osteoporosis population, while *Prevotella*, and *Enterobacterium* mainly appeared in non-osteoporosis patients (32). We also found a marked increase in the abundance of *Parabacteroides* and decrease in the abundance of *Prevotella* in the gut of GO patients after high-dose GC treatment. This also suggests that those changes may be one of the reasons for the occurrence of GC-induced osteoporosis. Through a variety of analytical methods, we found three intestinal bacteria (*Faecalibacterium*, *Streptococcus*, and *Prevotella*) which could be the biomarkers for distinguishing the two groups of before/after high-dose GC administration. LEfSe analysis also revealed that the abundances of *Faecalibacterium* and *Streptococcus* changed significantly after high-dose GC administration. Integrating previous studies and our findings suggests *Faecalibacterium*, *Streptococcus*, and *Prevotella* should be closely related to the occurrence and progression of GC-induced side effects such as hypertension, hyperglycemia, and osteoporosis.

SCFAs are important metabolites of the gut microbiota and its levels decreased significantly in many diseases, such as hypertension, hyperglycemia, and osteoporosis (33–36). One study showed SCFAs content was decreased in GC-induced obesity status (14). However, there is no literature on how the SCFAs change in the intestine of GO patients after high-dose GC administration. In our study, *Faecalibacterium* and *Prevotella*, two biomarkers which can produce SCFAs (37, 38), decreased significantly in GO patients’ gut after high-dose GC administration. Further functional predictions also revealed the decrease in the ability of propionate and butyrate metabolism in GO patients’ gut after high-dose GC administration. Finally, our qPCR results also showed that the ability to produce SCFAs, especially propionic acid and butyric acid, was reduced in GO patients after administering high-dose GCs. Several studies have confirmed that chronic delivery of the SCFAs via the drinking water lowered BP (39–41). These and other studies have identified several immune and non-immune mechanisms underlying this effect like regulating the function of immune cells such as T helper 17 (TH17) and T regulatory (Treg) cells (41, 42), and leading to cardiorenal transcriptomic changes by inhibiting histone deacetylases (43). SCFAs could ameliorate the progression of hyperglycemia via a variety of mechanisms, including maintaining the integrity of the intestinal epithelial barrier (44), promoting liver glycogen metabolism (45), regulating the function of mitochondria (46), as well as promoting the release of Glucagon-like Peptide-1 (GLP-1) and peptide YY by activating GPR43 (47). Studies have also found SCFAs are regulators of osteocyte metabolism and bone mass. The mechanism of the protective effect of SCFAs on bone mass lies in that SCFAs can reduce the expression of osteoclast-associated genes, such as TRAF6 and NFATc1 (35), leading to the inhibition of osteoclast differentiation and the reduction of bone resorption, promote the production of serum IGF-1, which is an anabolic stimulus to the bone (48, 49), and affect the intestinal Treg cell response (49). In conclusion, the reduction of SCFAs in the gut may be one reason for the increased susceptibility to hypertension, hyperglycemia, and osteoporosis when long-term/high-dose GCs were used.

Aside from SCFAs, 5-HT in the gut is also closely related to the occurrence and development of osteoporosis. The 5-HT signal transduction system is considered to play an important regulatory role in bone development and maintenance (50). Bliziotes et al. reported that increased level of serotonin is associated with decreased bone mass in mice (51). Another study found that using synthetic molecular inhibitors to reduce 5-HT level can prevent bone loss caused by ovariectomy (OVX) (52). Our metabolomics results show that 5-HT levels significantly increased in GO patients’ gut after high-dose GCs, which may be one of the reasons why patients using high-dose GCs are prone to osteoporosis.

The main mechanisms of high-dose GC treatment for GO are anti-inflammatory, immunosuppressive, and inhibition of fibroblast activity, and it has been used in clinical practice for a long time. We were not studying the occurrence mechanism of high-dose GCs exerting its therapeutic effects through the gut microbiota but rather focusing on how the high-dose GCs affected the intestinal flora. Therefore, we did not focus on the therapeutic effects of high-dose GCs on GO patients. Our study revealed for the first time the specific compositional changes of gut microbiota and metabolites in GO patients after use of high-dose GCs, and found that these changes may be closely related to the occurrence of GC-induced side effects (such as hypertension, hyperglycemia, and osteoporosis). A growing number of studies have shown that gut microbiomes can combine with other factors, such as diet, lifestyle, and drugs, to improve body health under physiological and disease conditions. The mechanisms underlying the beneficial effect of gut microbiome are very complex and require further study. At present, restoring the balance of the intestinal flora is considered a treatment method for various diseases. Specifically, the balance of intestinal flora can be restored by changing dietary habits and supplementing probiotics or their metabolites, such as SCFAs, and dietary fiber, even flora transplantation. Our work provides new ideas for the prevention and treatment of GC-induced side effects by restoring gut microbiota balance, though further studies are needed.

There are several limitations of this study. First, we included only 20 GO patients in this study. The small sample size may result in limitations in the statistical significance and generalizability of the findings, particularly in multifactorial analyses such as microbiome composition and metabolomic data analyses. These analyses are often significantly influenced by individual variability and complexity, necessitating a larger sample size to enhance the robustness of the results. Furthermore, we have clarified the scope of the study’s findings, emphasizing that small-sample studies can serve as exploratory research, but larger-scale validation trials are needed in the future to support our conclusions. Second, although we found differential bacteria in the intestine of GO patients after administering high-dose GCs, we did not further verify how high-dose GCs caused these bacteria to alter. Third, we have demonstrated by qPCR that the ability to produce SCFAs in the intestine of GO patients after high-dose GCs decreased, but we have not been able to directly detect changes of SCFAs in feces. Fourth, although our sequencing and metabolic data suggest that changes of gut microbiota and metabolites after high-dose GC treatment may lead to hypertension, hyperglycemia, and osteoporosis, further mechanistic studies are necessary for the understanding of some pathogenetic processes of the microbiota composition and function in GO patients after high-dose GC administration.

### Conclusion

In summary, gut microbiota dysbiosis in GO patients after high-dose GC administration is characterized by an increase in the abundance of *Streptococcus* and a decrease in *Faecalibacterium* and *Prevotella*, which may play an important role in GC-induced side effects. Furthermore, the reduction of SCFAs and the rise of 5-HT in gut may be reasons for the increased susceptibility to hypertension, hyperglycemia, and osteoporosis when long-term/high-dose GCs were used. Our work provides new ideas for the prevention and treatment of GC-induced side effects.

## Data Availability

The data that support the findings of the present study are available from the corresponding author on reasonable request. The 16S rRNA sequencing raw data is available at https://doi.org/10.6084/m9.figshare.28824032.v1.

## SUPPLEMENTAL MATERIAL

Supplemental material spectrum.02467-24-s0001.pdf Fig. S1 and S2; Tables S1 and S2.

ASM does not own the copyrights to Supplemental Material that may be linked to, or accessed through, an article. The authors have granted ASM a non-exclusive, world-wide license to publish the Supplemental Material files. Please contact the corresponding author directly for reuse.
