# Supplementary material for: Effects of high-dose glucocorticoids on gut microbiota in the treatment of Graves’ ophthalmopathy
Source: Microbiol Spectr. 2025 Apr 22;13(6):e02467-24. doi: 10.1128/spectrum.02467-24 (PMC12131860; doi:10.1128/spectrum.02467-24)
Supplement: Supplemental material — Fig. S1 and S2; Tables S1 and S2. [file spectrum.02467-24-s0001.pdf]

Figure S1

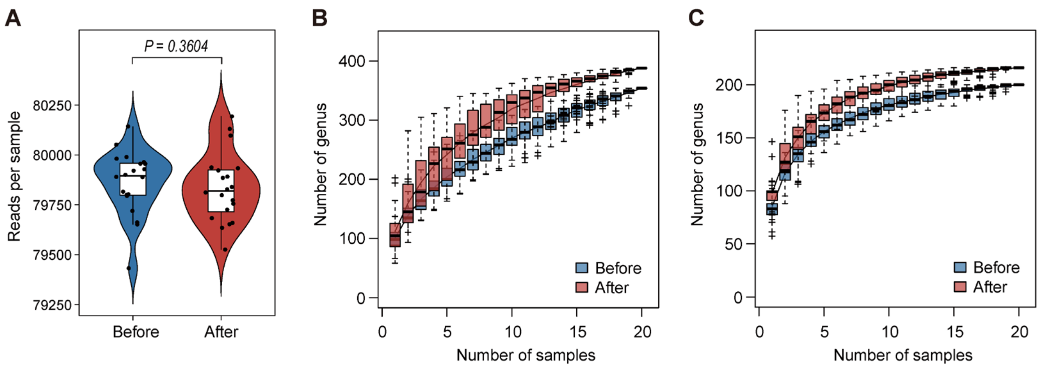

Figure S1: (A) Comparison of the 16S rRNA sequencing depth of stool samples from GO patients before administering high-dose GCs (Before, n=20) and after administering high-dose GCs (After, n=20). The paired samples Wilcoxon test was used to detect significant changes. (B) Species accumulation curves by genus. (C) Species accumulation curves based on genera with an abundance greater than 0.001%. Before: GO patients before administering high-dose GCs; After: GO patients after administering high-dose GCs; ns: not significant. n_{Before}=20; n_{After}=20.

Figure S2

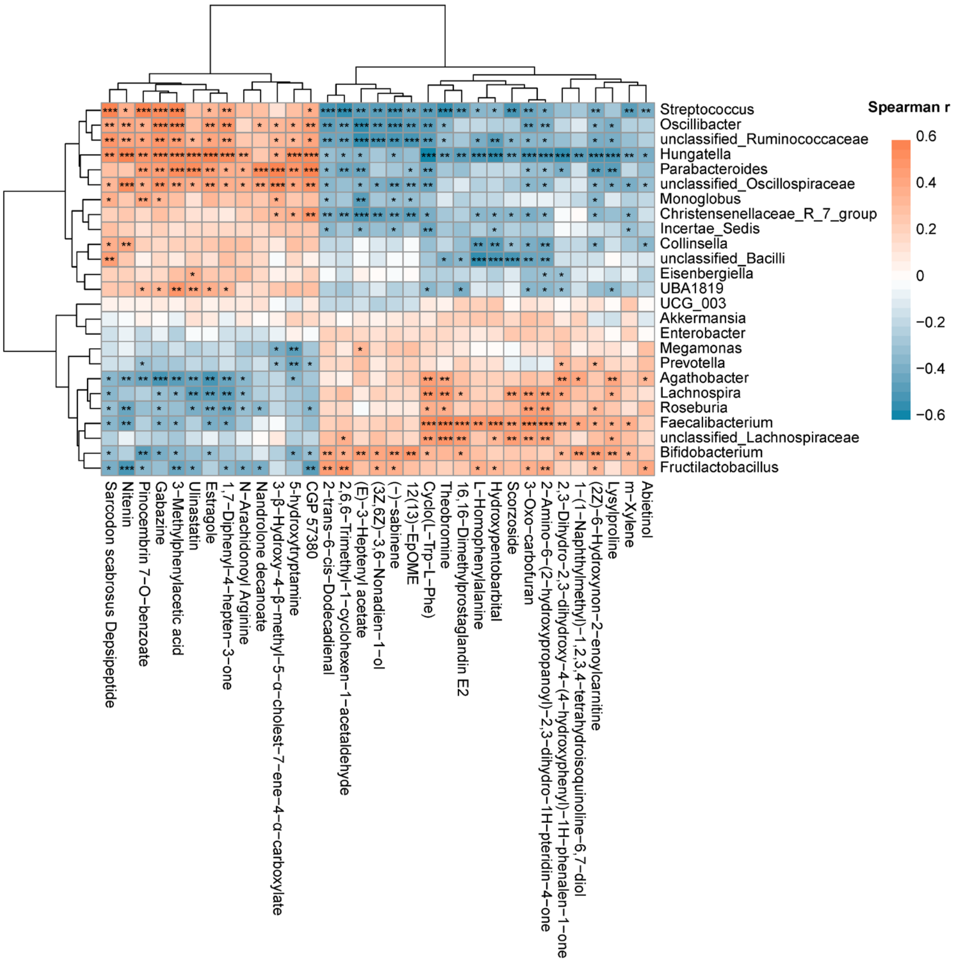

Figure S2: Heatmap showing the Spearman correlations between significantly differentially abundant microbial genera and differentially abundant metabolites. *: P< 0.05; **: P< 0.01; ***: P< 0.001.

Table S1. PCR Primers used in this study

Target group/gene	Primers	Primer sequence (5′ ⟶ 3′)
Total bacteria	Univ 337F	ACTCCTACGGGAGGCAGCA
	Univ 806R	GGACTACHVGGGTWTCTAAT
Total bacteria	Univ 337F	ACTCCTACGGGAGGCAGCAGT
	Univ 518R	GTATTACCGCGGCTGCTGGCAC
Firmicutes	pFirmF	GGAGYATGTGGTTTAATTCGAAGCA
	pFirmR	AGCTGACGACAACCATGCAC

Bacteroidetes	pBactF	CCGGAWTYATTGGGTTTAAAGGG
	pBactR	GGTAAGGTTCCTCGCGTA
Butyryl-CoA transferase	BCoATscrF	GCIGAICATTTCACITGGAAYWSITGGCAYATG
	BCoATscrR	CCTGCCTTTGCAATRTCIACRAANGC
Lactoyl-CoA dehydratase	lcdAF	CTGGTGTGCTGGWSIGCIWSIGTIGCNCC
	lcdAR	CAGATAGGTCCAIAYIGCDATNCCYTCCCA
Propionaldehyde dehydrogenase	pduPF	GTGGATGARACIGGIATGGGNAAYGTNGG
	pduPR	CAATAGCCYTCICCICCRAAICCIADNGC
Methylmalonyl-CoA decarboxylase	mmdAF	AATGACTCGGGIGGIGCIMGNATHCARGA
	mmdAR	GATTGTTACYTTIGGIACNGTNGCYTC

Table S2. Demographic characteristics of GO patients.

Sample	Sex (M/F)	Age (Y)
GO1	M	31
GO2	M	35
GO3	F	29
GO4	M	33
GO5	F	40
GO6	F	35
GO7	F	32
GO8	M	36
GO9	F	41
GO10	M	34
GO11	M	31
GO12	M	35
GO13	F	40
GO14	F	26

GO15	M	24
GO16	F	30
GO17	M	31
GO18	M	45
GO19	F	30
GO20	F	32

GO patients, Graves' ophthalmopathy patients; M, male; F, female; Y, year.
